# Concentrations of PCDD/Fs in Human Blood: A Review of Data from the Current Decade

**DOI:** 10.3390/ijerph16193566

**Published:** 2019-09-24

**Authors:** Montse Marquès, Jose L. Domingo

**Affiliations:** Laboratory of Toxicology and Environmental Health, School of Medicine, IISPV, Universitat Rovira i Virgili, Sant Llorenç 21, 43201 Reus, Catalonia, Spain

**Keywords:** polychlorinated dibenzo-*p*-dioxins (PCDDs), polychlorinated dibenzo-*p*-furans (PCDFs), human exposure, blood samples, dietary intake, risks

## Abstract

Polychlorinated dibenzo-*p*-dioxins and polychlorinated dibenzo-*p*-furans (PCDD/Fs) are environmental pollutants with great persistence, the capacity of bioaccumulation, and well known important toxic effects in humans and animals. Incinerators of hazardous, municipal and medical waste, chlorine bleaching of paper pulp, cement plants, and the traffic of motor vehicles are the most frequent emission sources of these compounds. The diet, followed at a great distance by inhalation, is generally the main way of human exposure to PCDD/Fs. Human biomonitoring is of great importance to prevent potential adverse effects derived from exposure to chemicals such as PCDD/Fs. In relation to this, blood is among the most used biological monitors. In the current review, we have summarized the recent information (2000–2009) published in the scientific literature (databases: Scopus and PubMed) on the concentrations of PCDD/Fs in blood samples of non-occupationally exposed populations, as well as in some groups of occupationally exposed individuals. We have revised a number of studies conducted in various African, American, Asian and European countries, and Australia. Unfortunately, the information is quite limited. No data are available for most countries over the world. Based on the results here reviewed, where available, the current health risks for the general populations do not seem to be of concern. Moreover, taking into account the important reductions observed in the levels of PCDD/Fs in foodstuffs, new decreases in the concentrations of PCDD/Fs in blood—and other biological tissues—are very probable in the immediate years.

## 1. Introduction

Polychlorinated dibenzo-p-dioxins and polychlorinated dibenzo-p-furans (PCDD/Fs) are chemical contaminants, whose main characteristics are great persistence, the capability to undergo long-range atmospheric transport, capacity of bioaccumulation, as well as their important toxic effects in humans and animals [1,2,3]. Although PCDD/Fs can be released in natural processes such as volcanoes and forest fires, these compounds are always unwanted by-products. Their environmental presence is mainly due to emission of industrial processes: incineration of hazardous, municipal and medical wastes, chlorine bleaching of paper pulp, cement plants and smelting [4,5,6,7]. However, it is well known that the primary source of human exposure to PCDD/Fs in the diet [8].

PCDD/Fs are currently included in the called “dirty dozen”, a group of dangerous persistent organic pollutants (POPs). Once in the body, PCDD/Fs are slowly eliminated and may elicit toxic effects including adverse reproductive effects, neurodevelopmental impairment, damage to the immune system, and endocrine disruption [9,10,11,12]. Moreover, the possibility that they can also cause cancer is especially worring. With respect to the potential carcinogenicity of PCDD/Fs, the congener 2,3,7,8-tetrachlorodibenzo-p-dioxin (TCDD) was evaluated by the International Agency for Research on Cancer (IARC) in 1997 and 2012, is currently classified as a “known human carcinogen” [13].

Due to the potential toxicity of PCDD/Fs and their frequent presence at different environmental concentrations, in recent decades human biomonitoring of PCDD/Fs has been an important issue to assess the risks of these chemicals on human health. The concentrations of PCDD/Fs have been determined in tissues such as kidney, liver, lung, pancreas and adipose tissue [14,15], but most biomonitoring studies have been conducted using blood and breast milk. Since PCDD/Fs are fat-soluble, both tissues are especially appropriate as biomonitors.

In recent years, the environmental levels of PCDD/Fs have followed a continuous reduction in most industrialized countries. As a direct consequence of these reductions, the dietary intake of PCDD/Fs—the main way of human exposure—by the general population has also dramatically diminished. For example, our area of residence, among other potential sources of PCDD/F emissions, such as a heavy traffic, counts with a hazardous waste and a municipal solid waste incinerators since more than two decades, where a spectacular significant decrease from 210 pg I-TEQ/day, in 1998 to 8.54 pg WHO-TEQ/day in 2018, has been recently noticed [16].

In this paper, we present an overview of the latest information on human exposure to PCDD/Fs, which is based on measuring the concentrations of these compounds in blood samples. In this same decade, Consonni et al. [17] published a comprehensive worldwide literature review -covering the period 1989–2010—of blood levels of dioxins and dioxin-like compounds in non-exposed adult general populations. PubMed (https://www.ncbi.nlm.nih.gov/pubmed) and Scopus (https://www.scopus.com/search/) were used as databases, with the search terms “dioxins, human exposure, blood” and “PCDDs, PCDFs, human blood”. Moreover, a complementary search was carried out by using the search terms: “dioxins, blood, various specific countries (China, USA, Germany, UK, etc)” in order not to lose any relevant information. Publications from regulatory organizations and other authorities, as well as gray literature available through the Internet, were intentionally excluded. This paper has been focused only on scientific publications. The period of the review covered between January 1, 2010 and August 7, 2019. Although only scientific publications within this period were included, some of the revised data may be referred to studies on dioxins in blood samples collected or analyzed in previous years. Next, we summarize the available information on the concentrations of PCDFFs in human blood of general populations of a number of countries (Table 1). We have also included data on workers at different facilities, who could be potentially exposed to PCDD/Fs.

## 2. Concentrations of PCDD/Fs in a Number of Countries

### 2.1. EUROPE

#### 2.1.1. Italy

Consonni et al. [17] published a comprehensive worldwide review of literature on human concentrations in blood of PCDD/Fs, dioxin-like compounds and TEQ levels in non-directly exposed populations of 26 countries in four continents. According to these authors, at that time no data on studies performed in Africa and Central or South America was available in the scientific literature. Eight of the studies included in the review were conducted in Italy, being all of them carried out in previous decades. Among these, some studies of special interest were those performed by Baccarelli et al. [50,51,52] regarding the effects of the Seveso Disaster (July 10, 1976). The Seveso accident caused a severe exposure of the population to 2,3,7,8-TCDD. According to Consonni et al. [18], none of the other PCDD/F congeners analyzed showed variation across the three zones in which the population was divided: A (very high contamination), B (high contamination) and R (low contamination). The median human plasma levels of 2,3,7,8-TCDD reported by Consonni et al. [18] were the following: zone A (73.3 pg TEQ/g fat), zone B (12.4 pg TEQ/g fat), and residents in zone R, the reference zone (5.5 pg TEQ/g fat).

#### 2.1.2. Belgium

Delvaux et al. [19] investigated the association between prenatal exposure to endocrine-disrupting chemicals (EDCs) and the body composition of 7–9 years-old Flemish children. The selected EDCs were cadmium, Polychlorinated Biphenyls (PCBs), dioxins, dichlorodiphenyldichloroethylene (p,p’-DDE) and hexachlorobenzene (HCB), whose concentrations were analyzed in cord blood/plasma. Prenatal exposure to dioxins was associated neither with body mass index (BMI) nor with weight at the age of 7 to 9 years. The median concentrations of PCDD/Fs in plasma were 0.05 and 0.04 pg CALUX -TEQ/L for boys and girls, respectively, being 0.05 pg CALUX-TEQ/L, for both boys and girls, who were not involved in the study. In the previous decade, a survey on the concentrations of PCDD/Fs in plasma of a Belgian population before and after the 1999 Belgian PCB/dioxin incident had been conducted by Debacker et al. [20]. It was reported that the total levels of dioxins in plasma significantly decreased between 1998 and 2000 (geometric means: from 445 to 417 pg/g fat). However, no significant differences between the total plasma dioxins were noticed when octachlorodibenzodioxin (OCDD) concentrations were excluded.

#### 2.1.3. Germany

Fromme et al. [21] determined the concentrations of PCDD/Fs and other environmental pollutants in blood samples from 70 subjects (4–76 years old), who lived at distances between 100 and 1000 m from a reclamation plant located in a rural area in Southern Germany. The median concentration was 4.5 pg WHO-TEQ/g fat weight. The dominant congener was OCDD, followed by 1,2,3,4,6,7,8-Heptachlorodibenzo-*p*-dioxin (1,2,3,4,6,7,8-HpCDD), 1,2,3,4,6,7,8-Heptachlorodibenzo-*p*-dioxin (1,2,3,4,6,7,8-HpCDD), 1,2,3,6,7,8-Hexaclhorodibenzo-*p*-dioxin (1,2,3,6,7,8-HxCDD) and 2,3,7,8-Pentachlorodibenzo-*p*-furan (2,3,7,8-PeCDF). This median concentration was lower than that obtained in a previous study of the same research group, with a median of 10.1 pg/g WHO-TEQ fat [53], which corresponded to an adult population of 50 healthy subjects living in Munich or nearby, with no past or present occupational exposure.

In a subsequent study conducted in the same laboratory [54], plasma samples were collected from the Bavarian Red Cross Blood Donation Service (Munich and surrounding areas) from 42 randomly selected subjects (20–68 years old). The median concentration for PCDD/Fs was 6.2 pg WHO-TEQ/g fat, in the same line than that found in the previous study [21]. Again, the PCDD/F congener profile was dominated by OCDD, followed by 1,2,3,4,6,7,8-HpCDD and 1,2,3,6,7,8HxCDD. In contrast, the concentration of 2,3,7,8-TCDD, the most toxic congener, was rather low.

#### 2.1.4. Spain

In Spain, most data on the concentrations of PCDD/Fs in human plasma have been obtained in our lab (Tarragona County, Catalonia). It is due to the fact that in 1999 started regular operations a new-and to date still the only one in Spain-hazardous waste incinerator (HWI), which is located in Constantí (Tarragona County). Due to the concern raised by that facility, a surveillance program was already established during the period of construction of the HWI. The baseline/background concentrations of metals and PCDD/Fs were determined in environmental (soils and herbage) samples, as well as in samples of various biological tissues of subjects living in the vicinity of the new facility. Blood was one of the tissues used for biomonitoring both metals and PCDD/Fs. In the baseline (1998) study, we found an average concentration of PCDD/Fs in plasma of 27.0 F;pg I-TEQ/g fat [22]. A continued and significant decrease in the plasma levels of PCDD/Fs have been noticed in subsequent surveys included in the surveillance program. Thus, in 2002 [23], 2007 [24] and 2012 [25], the mean PCDD/F concentrations in plasma of subjects living in the neighborhood of the HWI were 15.7, 9.4, F;and 6.18 pg I-TEQ/g fat, respectively. In turn, the results of the last study conducted in 2018 [26] showed a mean level of 6.79 pg I-TEQ/g fat, which was very similar to that found in 2012 (6.18 F;pg I-TEQ/g fat) [25]. Once again, OCDD was the predominant congener, while the lowest concentrations corresponded to 2,3,7,8-TCDD and 1,2,3,4,7,8,9-Heptachlorodibenzo-*p*-furan (1,2,3,4,7,8,9-HpCDF). Interestingly, we have noticed that the continued reduction in the PCDD/F levels in plasma is in accordance, and runs in parallel, with the decreasing trend also observed in the dietary intake of PCDD/Fs by that same population, which diminished from 210.1 F;pg I-TEQ/day in the baseline study [55] to 8.54 F;pg WHO-TEQ/day in the last (2018) study [16]. In the dietary intake studies conducted in 2002, 2006 and 2012 [56,57,58], the dietary intakes of PCDD/Fs by the population living in the area under potential influence of the HWI were 63.8, 27.8 and 33.1 pg WHO-TEQ/day, respectively. It means a spectacular decrease in the exposure to PCDD/Fs through the daily dietary intake since the initial 210.1 pg I-TEQ to the recently estimated of 8.54 pg WHO-TEQ [16].

In addition to the surveillance program for the general population of the area, a specific program for the workers of the HWI has been also carried out. The concentrations of various metals and organochlorinated substances in urine and blood samples of workers at the HWI have been periodically determined. The levels of PCDD/Fs in plasma samples are included in this program. In the baseline study [59] the mean concentrations of the 28 workers participating in that survey were 26.7 ng I-TEQ/kg fat. In subsequent studies, the mean PCDD/F levels have continuously decreased in parallel to the reductions also observed in the general population of the area [26], which in turn have been mainly attributed to the considerable decrease in the concentrations of PCDD/Fs in the most consumed foodstuffs in the area [16]. These are mean concentrations (ng I-TEQ/kg fat) of PCDD/Fs in pooled plasma samples of the workers at the HWI: 16.9 (2000), 10.3 (2002), 10.4 (2004), 5.5 (2008), 4.4 (2010,) and 4.6 (2011) [60,61,62,63,64].

In Biscay, Basque Country, Zubero et al. [27,28,29] have examined the evolution of the levels of PCDD/Fs in serum samples of an adult non-occupationally population living in the neighborhood -or far away- from the municipal solid waste incinerator (MSWI) of Bilbao, which started regular operations in 2005. Sampling (pooled samples) was performed in 2006 [27], 2008 [28] and 2013 [29]. The mean PCDD/F concentrations were 23.45, 23.60, and 4.67 pg WHO-TEQ/g fat for the samples collected in 2006, 2008 and 2013, respectively. The very important reduction with time is in total agreement with the above decrease also found in the HWI of Constantí, Tarragona County.

On the other hand, between 1995 and 2012, Parera et al. [30] monitored the concentrations of PCDD/Fs in blood samples from general populations considered exposed and non-exposed to the influence of the emissions of an MSWI in Mataró (Catalonia). No differences in blood levels of PCDD/Fs were noticed between subjects considered exposed (14.2 and 13.1 pg WHO-TEQ/g fat, in 1995 and 2012, respectively) and non-exposed (14.5–19.8 and 13.1–13.8 pg WHO-TEQ/g fat, in 1995 and 2012, respectively) to the emissions of the MSWI. These results suggest once again that the main route of human exposure to PCDD/Fs in the diet.

#### 2.1.5. Sweden

Salihovic et al. [31], measured the concentrations of a number of POPs in plasma samples of 1016 elderly subjects included in the Prospective Investigation of the Vasculature in Uppsala Seniors (PIVUS). Among these POPs, only one dioxin was included, OCDD, which is the congener most frequently detected. The median OCDD levels were 376 and 465 ng/g fat (0.376 and 0.465 I-TEQ ng/g fat), for men and women, respectively. This same research group again determined the POP levels –including also OCDD-to establish the relationship between the concentration of these POPs in blood and oxidative stress [65], as well as obesity [66,67]. Recently, Stubleski et al. [32] analyzed the plasma concentrations of a number of Cl/Br POPs in men and women (70–75 years old) participating in the PIVUS study. The levels of OCDD, the only analyzed dioxin, were below the detection limit in all samples.

### 2.2. ASIA

#### 2.2.1. China

There are no recent data on the levels of PCDD/Fs in the blood of populations who are not exposed to potential emissions of these compounds of their derivatives. However, there are some recent results regarding potentially exposed individuals. For example, Chen et al. [68] analyzed the concentrations of 17 PCDD/F congeners and 12 non-ortho and mono-ortho dioxin-like PCBs in the blood of 30 non-occupationally childbearing-aged women living near a chemical (Dagu Company) plant in Tianjin (China), which had been producing chlorinated pesticides during almost 50 years. The main purpose of that study was to determine whether the childbearing-aged residents living near the facility had a greater exposure risk. The concentrations of PCDD/Fs plus PCBs were between 16.43 and 55.29 pg WHO-TEQ/g fat (mean 62.5), being the contributions of PCDDs and PCDFs to the total TEQ value, 56.72%, and 34.44%, respectively. Total WHO-TEQ was approximately ten-fold higher in the participants living near the plant than in those living farther away. OCDD was the predominant congener, followed by 1,2,3,6,7,8-HxCDD and 1,2,3,4,6,7,8-HpCDD, while concentrations of 2,3,7,8-TCDD, were particularly low. In a recent study, Yu et al. [69] analyzed the concentrations of 17 PCDD/F and 12 dioxin-like (dl)-PCB congener profiles in 24 umbilical cord serum samples from pregnant women living near the Dagu Chemical Company (Tianjin, China). The aim of that study was to establish the trend of change of dioxins in local pregnant women, as well as to assess the health risks related to fetal exposure. The concentrations of ∑(PCDD/Fs + dl-PCBs) were between 476 and 8307 pg/g fat (mean: 3037 pg/g fat). The mean WHO-TEQ for PCDD/Fs was 14.0 pg/g fat.

On the other hand, Xu et al. [33] carried out a study in which the blood concentrations of PCDD/Fs were determined in school-age children living near an MSWI in Mainland. The potential associations of these concentrations with the children’s genetic, epigenetic, hormonal, immunological, and hematological characteristics were assessed. The mean blood levels of ΣPCDD/Fs and TEQ-ΣPCDD/Fs were significantly higher in the potentially exposed group than in the control group: 3.40 vs. 2.77 F;pg/g wet weight, and 0.40 vs. 0.28 F;pg WHO-TEQ/g wet weight, respectively. It was concluded that although the diet is the main route of exposure to PCDD/Fs, children living near the MSWI might suffer genetic and epigenetic modifications, such as DNA damage or global DNA hypomethylation, due to the MSWI-emitted PCDD/Fs.

#### 2.2.2. Taiwan

In Taiwan, the most recent data on the concentrations of PCDD/Fs in blood correspond to the investigation conducted to examine the potential association between the PCDD/F levels in blood and diabetes mellitus [34]. The study was performed in an endemic area of exposure. A total of 2898 subjects participated in the study. Among them, 1143 had dioxin levels in serum between 20 and 63 pg WHO-TEQ/g fat, while in 284, the PCDD/F concentrations were, at least, 64 pg WHO-TEQ/g fat. There were 339 cases of diabetes mellitus (23.8%) showing a high PCDD/F exposure, ≥20 pg WHO-TEQ/g fat. The authors concluded that exposure to PCDD/Fs was a risk factor for diabetes mellitus, independently of the age and body mass index. In another Taiwanese study conducted in the previous decade, Hsu et al. [35] had reported a median concentration of PCDD/Fs in 251 serum samples collected from the general population of 11.5 pg WHO-TEQ/g fat (range 4.92–26.7). The differences with respect to the study by Huang et al. [34] are evident.

#### 2.2.3. Korea

In the current decade, the only available data on the levels of PCDD/Fs in human blood samples correspond to 30 incineration workers, in whose plasma the mean toxic equivalent concentration was 11.32 pg/g fat. In turn, mean TEQ levels in the low and high exposed groups were 6.02 and 17.80 pg/g fat, respectively [70].

#### 2.2.4. Vietnam

Van Luong et al. [36] determined the association between exposure to PCDD/Fs and the concentrations in blood of reproductive hormones in 42 men living around the Bien Hoa airbase, which is considered the largest and most dioxin-contaminated area of Vietnam. The geometric mean of sum TEQ of PCDD/Fs was 34.0 pg/g fat, an elevated value, which, according to the authors might increase the levels of prolactin and decrease those of total testosterone in men.

#### 2.2.5. Japan

In the past decade, Todaka et al. [37] measured the levels of PCDD/Fs in blood samples collected from 195 pregnant women in Sapporo City. The arithmetic mean total TEQ concentration of PCDD/Fs was 17.4 pg/g fat. Subsequently, the same research group [38] measured the concentrations of PCDD/Fs (and also non-dioxin-like PCBs) in paired samples of blood and breast milk collected from 30 primiparous and 30 secundiparous mothers living in Sapporo City, Hokkaido Prefecture, Japan. The mean concentrations of PCDD/Fs in blood were 12.3 and 9.8 pg TEQ/g fat, in primiparous and multiparous mothers, respectively [38]. Subsequently, the concentrations of these compounds were measured in paired samples of blood and breast milk collected from 89 primiparous mothers living in the same place [39]. The mean level of PCDD/Fs in blood of these 89 mothers plus the previous 30 primiparous [38] was 11.1 pg TEQ/g fat. In another study conducted by the same research group [40], the concentrations of PCDD/Fs were again determined in blood and breast milk samples collected from 67 secundiparous mothers in Sapporo City. The mean concentration of PCDD/Fs, in blood of these 97 secundiparous mothers plus the previous 30 secundiparous [38] was 9.1 pg TEQ/g fat.

Arisawa et al. [41] assessed the association between dietary patterns and the blood levels of PCDD/Fs in 1656 subjects from 90 different study areas of 30 prefectures of Japan. The median concentration of PCDD/Fs in the blood of that population was 16 pg TEQ/g fat. In accordance with the results of a number of studies conducted elsewhere, the dietary habits were positively correlated with the blood levels of PCDD/Fs. In that case, the frequent intake of seafood and alcoholic beverages were associated with a higher total TEQ of PCDD/Fs in blood. In parallel, and with the same subjects, the correlations of fish intake and plasma docosahexaenoic acid (DHA) levels with each PCDD/F congener in were examined [71]. Plasma concentrations levels of six PCDDs/Fs with 4–6 substituted chlorine atoms, but not HeptaCDD/F and OctaCDD, showed significant positive correlations with fish intake and plasma DHA concentrations in that Japanese population. Recently, Muzembo et al. [42] assessed the correlation between dioxin dietary intake and corresponding body burden in the Japanese population. Blood and food samples were collected. The median total TEQ in the blood of participants living in fishing villages was 11.0 (9.7–12.0) pg TEQ/g fat, a value relatively higher compared to those found in urban, 7.4 (5.8–8.8) pg TEQ/g fat, and agricultural/farming areas, 8.3 (7.5–9.1) pg TEQ/g fat. Median blood dioxins and dietary dioxin intake were approximately 41% lower compared with data obtained in the period 2002–2010 [42]. These results are in agreement with the recent conclusions of a review by Arisawa [72] on the associations of blood levels of dioxins with metabolic diseases, in which it was noticed that the blood levels of dioxins were decreasing, probably due to the reduction in the dietary intake of these pollutants.

Although to examine the effects of Yusho disease is not among the objectives of the present review, we would like to notice that in the current decade, various studies on this topic have been carried out. They have been focused on correlating the effects of Yusho disease on mothers and their descendants with the levels of PCDD/Fs and PCBs in the blood of these mothers [73,74,75]. An interesting review of studies conducted by the Yusho Group was recently published [76].

### 2.3. AMERICA

#### 2.3.1. USA

Horii et al. [43] reported congener-specific concentrations of various organochlorinated environmental pollutants, including PCDDs and PCDFs, in retrospectively sampled blood plasma from New York State personnel (43 subjects), who had responded to the World Trade Center (WTC) disaster of September 11, 2001. Four subgroups were established: More Dust Exposed (MDE), Less Dust Exposed (LDE), More Smoke Exposed (MSE) and Less Smoke Exposed (LSE). Mean concentrations of PCDDs were 1070, 223, 3690, and 732 pg/g fat, while the mean levels of PCDFs were 910, 1520, 230, and 117 pg/g fat, for the MSE, MDE, LSE, and LDE subgroups, respectively. Based also on the exposure to pollutants released in the WTC disaster, recently Kahn et al. [44] determined, more than 12 years later, the serum levels PCDD/Fs of youths present in lower Manhattan and aged < 8 years, on September 11, 2001. A matched comparison group was included in the study. The mean concentration in WTC Health Registry (WTCHR) participants (72.5 pg TEQ/g fat) was notably higher (> 7 times) than that observed in non-WTCHR participants (10.1 pg TEQ/g fat) [44]. To put these data in context, we have compared these results with concentrations of PCDD/Fs in serum samples collected during the 1999–2004 period, as part of the National Health and Nutrition Examination Survey (NHANES). Samples were analyzed from the 1999 to 2000, and 2001 to 2002 time periods. The results were 13.46 and 13.98 pg TEQ/g fat, respectively, being 11.39 pg TEQ/g fat in the 2003–2004 time period [45]

#### 2.3.2. Mexico

Rodriguez-Dozal et al. [46] measured the levels of several POPs s in samples of plasma of 240 pregnant women living in 10 Mexican cities. PCDDs and PCDFs were only available for two composite samples per city. The total levels of PCDDs and PCDFs were 5.0 and 1.3 pg TEQ/g fat, respectively. The levels of 2,3,7,8 TCDD-TEQ in the composite plasma samples were very similar in all cities, with the exception of Coatzacoalcos, where the PCDD-TEQ concentrations were more than the double of the levels found in other cities.

### 2.4. AFRICA

#### 2.4.1. South Africa

In order to investigate whether burning solid biofuel to cook food and heat the homes could lead to elevated concentrations of PCDD/Fs and PCBs, Pieters and Focant [47] determined the serum concentrations of PCDD/Fs in a South African population. This was the first study in the country that included more than 100 participants. Mean serum fat content was 6.9 pg TEQ/g fat, a comparable value to unexposed populations in other parts of the world.

#### 2.4.2. Ghana

Wittsiepe et al. [77] measured the levels of PCDD/Fs in blood samples of workers from the Agbogbloshie e-waste dumps/recycling site (EWRS) in Accra, as well as those of controls from the surrounding area, without exposure to e-waste recycling. In subjects of the EWRS-exposed group, the median concentration of PCDD/Fs was 6.18 (range: 2.1–42.7 pg WHO-TEQ/g fat), a level significantly higher than that of the individuals in the control group: 4.60 (range: 1.6–11.6 pg WHO-TEQ/g fat). A recent review on the state of POPs in Ghana reported concerns of an absence of human biomonitoring studies on PCDD/Fs, dioxin-like compounds, as well as emerging contaminants [78]. In relation to this, recently Bruce-Vanderpuije et al. [48] have provided baseline blood concentrations of 17 congeners of PCDD/Fs, among other environmental contaminants, which were obtained of 34 primiparous Ghanaians living in the municipalities of Accra (near to the Agbogbloshie e-waste site) and Tema. The total mean concentrations for PCDD/Fs in Tema and Accra were 52.6 F; and 71.8 F;pg/g F;fat, respectively. The difference in total TEQ was statistically significant (1.55 vs. 2.64 pg WHO-TEQ/g fat).

#### 2.4.3. Other Data from African Countries

Recently, Pius et al. [79] have published an overview of the available information on the release of PCDD/Fs (and also dl-PCBs) and other critical data relevant to their monitoring in Africa since the existence of the Stockholm Convention. That review summarizes data on human exposure including PCDD/F levels in blood samples (which have been above discussed), human milk, and environmental samples such as soil and sediments. It is also focused on the annual releases of PCDD/Fs (g TEQ) of various African countries, the concentrations in a number of foodstuffs, and the levels in passive air samples. Finally, the review also examined available methods of analysis for PCDD/Fs (and dl-PCBs), while an analysis of existing gaps was also undertaken. Another recent paper has also reviewed the scientific literature for the last two decades with an emphasis on levels, toxic equivalencies, and sources of PCDD/Fs (and dioxin-like PCBs) in Africa [3]. It was noticed that little data were available about the levels and sources of PCDD/Fs in environmental and biological samples from Africa.

### 2.5. AUSTRALIA

Staff et al. [49] assessed whether local residents in Sidney were exposed to significant amounts of dioxins from a large urban remediation process. The levels of PCDD/Fs in blood were used as biomonitors. The mean concentrations were 15.0 and 13.1 pg WHO-TEQ/g fat, for local residents and for control individuals, respectively. It meant decreases between both the local resident and control groups over the remediation period by 1.9 and 2.1 pg TEQ/g fat, respectively. However, the overall pattern of TEQ decline is most probably due to the general decline in overall national dietary exposures of PCDD/Fs than because of the local remediation process.

## 3. Summary and Conclusions

The levels of PCDD/Fs in human blood samples reviewed in the current paper differ considerably among the countries for which studies have been recently conducted. It is important to note that we have not found recent information corresponding to many highly industrialized countries. Among the EU countries, only data on Belgium, Germany, Italy, the Netherlands, Spain, and Sweden were found in the databases, Scopus and PubMed. No information on the United Kingdom and Russia, for example, is available. Data corresponding to Canada and South American countries is non-existent, while data from African countries are scarce. Anyway, in general terms, and based on the current information about the PCDD/F levels in the blood, the health risks for the general non-occupationally exposed populations do not seem to be of concern. However, it must be noticed that according to the IARC, dioxins can cause cancer, and therefore, as for any carcinogenic substance, there are no safe concentrations of PCDD/Fs. In this sense, Lakind et al. [45] introduced the Biomonitoring Elements (BE), which take into account toxicological data of PCDD/Fs, pharmacokinetics of PCDD/Fs in humans, and the appropriate uncertainty factor components to the toxicological point of departure. Thus, BE values can be used as screening tools to identify whether the measured population concentrations are in the region of low, medium, or high priority for risk assessment follow-up. Hence, their application might be valuable to offer an important context to the blood TEQ values hereby reported.

The environmental emissions of PCDD/Fs have been continuously decreasing in recent years. It is because some important sources of these emissions, such as waste incinerators, are subjected to rigorous controls. In addition, the use of diesel and other fossil fuels in motor vehicles is also significantly decreasing. Consequently, it is logical to expect less human environmental exposure to PCDD/Fs in the coming years.

The diet is the main route of exposure to PCDD/Fs. Therefore, a considerable reduction of the environmental levels of PCDD/FS should have a direct repercussion on the concentrations of these compounds in foodstuffs, and consequently in human exposure through the diet. Thus, new decreases in the concentrations of PCDD/Fs in blood and other biological tissues are very probable. However, it is important to fill the gaps still existing, especially conducting studies in those countries where there is no information on human exposure to PCDD/Fs, neither environmental nor through the diet.

## Figures and Tables

**Table 1:** Summary of the most relevant studies on the occurrence of PCDD/Fs in the blood of non-occupational exposed population.

Country	Subjects of Study	Sample	Concentration	Main Conclusion	Reference
**Italy**	Population potentially affected by Seveso accident	Plasma	Median levels of 2,3,7,8-TCDD: very high contaminated site = 73.3 pg TEQ/g fat high contaminated site = 12.4 pg TEQ/g fat reference zone = 5.5 pg TEQ/g fat	The Seveso accident caused a severe exposure of the population to 2,3,7,8-TCDD.	[18]
**Belgium**	Prenatal exposure to PCDD/Fs and association with body composition at 7–9 years	Plasma	Median concentration = 0.05 pg CALUX-TEQ/L (boys) Median concentration = 0.04 pg CALUX-TEQ/L (girls)	Prenatal exposure to dioxins was not associated with BMI	[19]
	1999 dioxin incident	Plasma	Geometric mean concentrations: 1998: 445 pg/g fat 2000: 417 pg/g fat	The total levels of dioxins in plasma significantly decreased between 1998 and 2000. However, no significant differences between the total plasma dioxins were noticed when OCDD concentrations were excluded	[20]
**Germany**	Population living in the surroundings of a reclamation plant	Plasma	Median concentration: 4.5 pg WHO-TEQ/g fat	This study did not exhibit elevated internal exposures. The results also hint further decreasing tendencies for PCDD/Fs in Germany and demonstrate that people in the vicinity of a reclamation plant with no indication of environmental contamination did not exhibit elevated internal exposures	[21]
	Population living in Munich and surrounding	Plasma	Median concentration: 10.1 pg WHO-TEQ/g fat		
	Population living in Munich and surrounding	Plasma	Median concentration s: 6.2 pg WHO-TEQ/g fat		
**Spain**	Population living in the surroundings of an HWI	Plasma	Mean concentrations: 1998: 27.0 pg I-TEQ/g fat 2002: 15.7 pg I-TEQ/g fat 2007: 9.4 pg I-TEQ/g fat 2012: 6.18 pg I-TEQ/g fat 2018: 6.79 pg I-TEQ/g fat	The continued reduction in the PCDD/F levels in plasma is in accordance, and runs in parallel, with the decreasing trend also observed in the dietary intake of PCDD/Fs	[22,23,24,25,26]
	Population living in the surroundings of an MSWI located in Bilbao	Plasma	Mean concentrations: 2006: 23.45 pg I-TEQ/g fat 2008: 23.60 pg I-TEQ/g fat 2013: 4.67 pg I-TEQ/g fat	Individuals living near to a solid waste MSWP did not have higher blood levels of OCs than those living further afield, and they decreased over time	[27,28,29]
	Population living in the surroundings of an MSWI located in Mataró	Plasma	Mean concentrations of the exposed population: 1995: 14.2 pg I-TEQ/g fat 2002: 13.1 pg I-TEQ/g fat	No differences in blood levels of PCDD/Fs were noticed between subjects considered exposed and non-exposed to the emissions of the MSWI, suggesting once again that the main route of human exposure to PCDD/Fs in the diet	[30]
			Range concentrations of the non-exposed population: 1995: 14.5-19.8 pg I-TEQ/g fat 2002: 13.1-13.8 pg I-TEQ/g fat		
**Sweeden**	OCDD levels in subjects of the Prospective Investigation of the Vasculature in Uppsala Seniors	Plasma	Mean concentrations between 2001–2004: 376 ng/g fat (men) 465 ng/g fat (women)	The concentrations of OCDD were found similar, or comparable, to other studies of non-occupationally exposed populations from Sweden and Europe. Levels of OCDD were found to be higher in women.	[31]
		Plasma	<LD	OCDD were below the detection limit for all samples and therefore excluded from the longitudinal evaluation	[32]
**China**	The mean blood levels of ΣPCDD/Fs in school-age children living near an MSWI	Blood	Exposed group: 3.40 pg/g wet weight 0.40 WHO-TEQ/g wet weight Control group: 2.77 F;pg/g wet weight 0.28 F;pg WHO-TEQ/g wet weight	The mean blood levels of ΣPCDD/Fs and TEQ-ΣPCDD/Fs were significantly higher in the potentially exposed group than in the control group. It was concluded that although the diet is the main route of exposure to PCDD/Fs, children living near the MSWI might suffer genetic and epigenetic modifications, such as DNA damage or global DNA hypomethylation, due to the MSWI-emitted PCDD/Fs.	[33]
**Taiwan**	Endemic area of exposure to PCDD/Fs	Serum	1143/2898 people: 20–63 pg WHO-TEQ/g fat	Exposure to PCDD/Fs was a risk factor for diabetes mellitus, independently of the age and body mass index	[34]
			284/2898 people: >64 pg WHO-TEQ/g fat		
			339 cases of diabetes mellitus (>20 pg WHO-TEQ/g fat)		
	General population	Serum	Median concentration = 11.5 pg WHO-TEQ/g fat (range 4.92–26.7)	The levels of PCDD/PCDFs increased by 0.16 WHO 1998-TEQ/g fat per year for subjects above the age of 30, but there was no evidence of any association between age and the levels for subjects below the age of 30 years. More research is needed to investigate the causes of the different trends in young and old subjects in Taiwan.	[35]
**Vietnam**	Men living around the Bien Hoa airbase	Blood	Geometric mean = 34.0 pg/g fat	Elevated dioxin concentrations in the blood of men living near the Bien Hoa airbase were found. Dioxin exposure may increase levels of prolactin and decreased levels of total testosterone in men.	[36]
**Japan**	Pregnant women from Sapporo city	Blood	Arithmetic mean = 17.4 pg/g fat	The total TEQ concentration of PCDD/Fs in pregnant women has decreased compared to past levels in Japan for the last several decades	[37]
	Primiparous and secundiparous mothers from Sapporo	Blood	Mean concentration: 12.3 pg TEQ/g fat (primiparous mother) 9.8 pg TEQ/g fat (multiparous mother)	The body burdens of PCDD/Fs in mothers from Sapporo City was lower than that recently reported in Japan. These lower TEQ levels obtained in the present study may indicate a reduction of the background levels both in the environment and in the food of Sapporo City over recent decades	[38]
	Primiparous mothers from Sapporo	Blood	Mean concentration = 11.1 pg TEQ/g fat	Statistically significant correlations were observed between maternal age and the total TEQ concentration of PCDD/Fs. The results obtained in the present study may provide useful information regarding the health risk of PCDD/Fs in children and aid in future epidemiological investigations of the effects of these compounds on children.	[39]
	Secundiparous mothers from Sapporo	Blood	Mean concentration = 9.1 pg TEQ/g fat	These data may provide important information regarding the health risk of these compounds in infants. In the future, the collection of these data from many more mothers is warranted. Further research must be undertaken in the context of epidemiological investigations to more accurately assess the effects of these compounds on children	[40]
	Subjects from 90 different study areas of 30 prefectures	Blood	Median concentration = 16 pg TEQ/g fat	The dietary habits were positively correlated with the blood levels of PCDD/Fs. In that case, the frequent intake of seafood and alcoholic beverages were associated with a higher total TEQ of PCDD/Fs in blood.	[41]
	General population	Blood	Median blood levels = 9.4 (8.8–9.9) pg TEQ/g fat	Median blood dioxins and dietary dioxin intake were approximately 41% lower compared with data obtained in the period 2002–2010	[42]
**USA**	New York State personnel responding to the WTC disaster of 11S collapse	Plasma	Mean concentrations of PCDDs: more smoke-exposed = 1070 pg/g fat more dust exposed = 223 pg/g fat less smoke exposed = 3690 pg/g fat less dust exposed = 732 pg/g fat. Mean levels of PCDFs more smoke-exposed = 910 pg/g fat more dust exposed = 1520 pg/g fat less smoke exposed= 230 pg/g fat less dust exposed = 117 pg/g fat	Plasma levels of PCDFs in more smoke exposure and more dust exposure groups were higher than the levels found for less smoke-and less dust-exposure groups, suggesting exposure of the WTC responders to PCDFs after the collapse of WTC. On the basis of TEQs, we conclude that PCDFs are the critical dioxin-like compounds in the more smoke-exposed/more dust exposed groups, whereas PCDDs are the critical contaminants in the less smoke-exposed/less dust exposed groups	[43]
	Youths present in lower Manhattan and aged < 8 years, on 11S collapse	Plasma	Mean concentration in WTC Health Registry (WTCHR) participants: 72.5 pg TEQ/g fat Mean concentration in non-WTCHR participants: 10.1 pg TEQ/g fat (>7 times than that observed in non-WTCHR participants)	Adolescents in lower Manhattan on the day of the WTC attack and exposed to particulate contamination from the WTC collapse had significantly elevated PCDD/F levels > 12 years later compared to a matched comparison group, driven by chronic home dust exposure rather than acute dust cloud exposure. PCDD/F and TEQ levels substantially exceeded those in similar-aged NHANES participants. Future studies are warranted to explore associations of PCDD/Fs with health and developmental outcomes among individuals exposed to the WTC disaster as children.	[44]
	General population	Plasma	Mean PCDD/Fs concentrations: 1999–2000: 13.46 pg TEQ/g fat 2001–2002: 13.98 pg TEQ/g fat 2003–2004: 11.39 pg TEQ/g fat	From 1999 to 2004, PCDD/F serum levels decreased by 56% for the 12-to 19-year-old group and by 38% for the 20-to 39-year olds. A slight nonsignificant decrease was observed for 40-to 59-year olds and a slight significant increase was found for 60-year-olds. Interpretation of the data across time is complicated by certain aspects of the data unique to the various sampling time periods, thus, caution should be exercised when evaluating trend information.	[45]
**Mexico**	Pregnant women living in 10 Mexican cities	Plasma	The total levels of PCDDs = 5.0 pg TEQ/g fat The total levels of PCDFs = 1.3 pg TEQ/g fat	The levels of 2,3,7,8 TCDD-TEQ in the composite plasma samples were very similar in all cities, with the exception of Coatzacoalcos, where the PCDD-TEQ concentrations were more than the double of the levels found in other cities. Although this study provides useful information on the variability of PCDD/Fs in specific populations and possible regional/local differences, these results cannot be generalized to the entire Mexican population because of differences in age, gender, sources of exposure and nonrandom nature of the sample.	[46]
**South Africa**	Population exposed to burning solid biofuel to cook	Serum	Mean concentration = 6.9 ± 3.3 pg/g fat. The females had higher serum levels of the PCDDs and PCDFs	The mean serum levels of PCDD/Fs for this rural population were comparable to unexposed human populations in other parts of the globe. Surprisingly, electricity (and gas and paraffin) users had significantly higher serum levels of PCDDs, PCDFs (pg/g fat, WHO-TEQ pg/g) and ΣPCDD/Fs. The role of gender was inconsistent in terms of the various compound categories, despite women having more body fat than men, they were not always the group with the highest burden of contaminants. The individuals who worked away from home seemed to be better off because they carried fewer pollutants	[47]
**Ghana**	Primiparous mothers from Accra and Tema	Serum	Mean concentrations in Tema were 52.6 F;pg/g F;fat and 1.55 pg WHO-TEQ/g fat Mean concentrations in Accra were 71.8 F;pg/g F;fat and 2.64 pg WHO-TEQ/g fat	Positive correlations were obtained for total dioxins concentrations with age and Body Mass Index. Dietary intake of seafood and dairy products had a strong influence on PCDD/F concentrations. Statistically significant differences were observed for dioxins in participants from Accra (in close proximity to Agbogbloshie e-waste site) and Tema.	[48]
**Australia**	Local residents in Sidney exposed to dioxins from a large urban remediation process	Blood	Mean concentrations: Locals residents = 15.0 pg WHO-TEQ/g fat Control individuals = 13.1 pg WHO-TEQ/g fat	There was a decrease between both the local resident and control groups over the remediation period	[49]

## Abbreviations

TEQ	Toxic Equivalents
CALUX	Chemically Activated LUciferase gene eXpression
